# Comparing internal versus external fixation for diaphyseal tibial and fibular fractures in skeletally immature dogs

**DOI:** 10.18849/ve.v9i1.677

**Published:** 2024-01-31

**Authors:** Jake Chitty+, Paul Aldridge+

**Keywords:** CANINE, DOGS, EXTERNAL FIXATOR, FIBULAR FRACTURE, FIXATION, FRACTURE HEALING, FRACTURE REPAIR, INTERNAL FIXATION, ORTHOPAEDICS, POSTOPERATIVE COMPLICATIONS, SKELETALLY IMMATURE CANINE, TIBIAL FRACTURE

## Abstract

**PICO question:**

In skeletally immature dogs with simple non-displaced diaphyseal tibial and fibular fractures does internal fixation compared with external fixation result in less postoperative complications and improved fracture healing?

**Clinical bottom line:**

**Category of research:**

Treatment.

**Number and type of study designs reviewed:**

There were no publications that answered the PICO question.

**Strength of evidence:**

None.

**Outcomes reported:**

Both external skeletal fixation and internal fixation are reported as techniques for diaphyseal tibial and fibular fracture management in companion animals, though no study has been reported to compare these techniques directly, or to report fracture healing and postoperative complications in skeletally immature dogs with non-displaced diaphyseal tibial fractures.

**Conclusion:**

Given the absence of evidence answering the PICO, choice and recommendation on treatment for non-displaced diaphyseal tibial and fibular fractures in skeletally immature dogs should be decided on personal experience and stabilisation methods available to the veterinarian such as external fixation or internal fixation. Both surgical techniques have been reported in skeletally mature and immature dogs with diaphyseal tibial fractures, but not specifically in skeletally immature patients with non-displaced diaphyseal tibial and fibular fractures. They are both applicable methods of fixation for tibial fractures in companion animals, though there is lacking evidence for which has more favourable outcomes for non-displaced diaphyseal tibial and fibular fractures in skeletally immature dogs as no studies have directly compared these stabilisation techniques.

## How to apply this evidence in practice

The application of evidence into practice should take into account multiple factors, not limited to: individual clinical expertise, patient’s circumstances and owners’ values, country, location or clinic where you work, the individual case in front of you, the availability of therapies and resources.

Knowledge Summaries are a resource to help reinforce or inform decision-making. They do not override the responsibility or judgement of the practitioner to do what is best for the animal in their care.

## Clinical scenario

An 8-week-old Labrador Retriever puppy presents to you a non-weight bearing lame of the right hind limb after falling off the sofa. Radiographs reveal a simple non-displaced transverse diaphyseal tibial and fibular fracture. The contralateral limb is unaffected. You have orthopaedic implants available at your clinic to offer external fixation or internal fixation and you wish to find evidence of which technique results in a lower rate of postoperative complications and improved fracture healing.

## The evidence

There was no evidence that addressed the PICO from a literature search.

## Summary of the evidence

There was no evidence that compared external fixation to internal fixation for skeletally immature canine patients with non-displaced diaphyseal tibial and fibular fractures. In view of the absence of this evidence, it is recommended that veterinarians should base treatment choice on available orthopaedic equipment at their clinic and their personal experience with fracture stabilisation methods they are comfortable using. They should acknowledge that both methods have potential risks and complications.

## Appraisal, application and reflection

There was no evidence that directly addressed the PICO question. Published studies have included skeletally immature dogs in case populations of tibial diaphyseal fractures but no studies have specifically compared these two fixation methods for skeletally immature dogs with non-displaced tibial fractures directly.

Tibial and fibular fractures are commonly encountered fractures in companion animals, making up 10–20% of all fractures (Hayashi & Kapatkin, 2018). No studies have compared outcomes between external skeletal fixation or internal plate fixation in skeletally immature dogs with non-displaced diaphyseal tibial and fibular fractures or reported the incidence of non-displaced diaphyseal tibial and fibular diaphyseal fractures in skeletally immature canine patients. Both external skeletal fixation (ESF) (Pettit, 1992; McCartney, 1998; Gül & Yanik, 2006; and Sherman et al., 2022) and internal plate fixation (IPF) (Haaland et al., 2009; and Beale & McCally, 2020) have been reported for treatment of tibial diaphyseal fractures in companion animals in the veterinary literature.

When it comes to choosing the fixation method for such fractures the veterinarian should consider their experience in internal and external fixation, their available materials for the methods, the potential risks and complications reported with each method of fixation which the veterinarian will need to be equipped to manage, and the practicalities for the patient and owner in the postoperative recovery period.

Even within these two methods of fixation, different techniques can be used. Internal fixation utilising plates and screws can be achieved via open reduction and internal fixation (ORIF) or minimally invasive techniques (MIT) (Beale & McCally, 2020). The availability of advanced imaging such as fluoroscopy to help guide MITs is still limited outside referral veterinary practice, often limiting MITs to a referral veterinary practice setting where intra-operative imaging modalities are available. A study by Boero Baroncelli et al. (2012) found no significant difference in radiographic healing between open reduction versus minimally invasive approaches for internal fixation of canine tibiae. Only four cases included in this study were skeletally immature dogs and the total case number was 16 cases (eight MIT compared to eight ORIF) warranting caution in the interpretation of these results.

Different materials and configurations of ESF can be constructed for fracture fixation. The different external fixator constructs that have been reported to successfully treat diaphyseal tibial fractures (McCartney, 1998; Aydin et al., 2022; and Sherman et al., 2022), may have differing learning curves and may require theoretical and practical courses prior to their use in practice, which the veterinarian may wish to consider prior to obtaining and using this instrumentation.

It is important that the veterinarian is aware of the possible complications associated with ESF and IPF when deciding treatment options as they will need to be equipped to manage these complications if they occur.

A case series by Aronsohn & Burk (2009) evaluated type 1a external skeletal fixator (ESF) for tibial fracture repair in five skeletally immature canine patients (age range 12–23 weeks) for treatment of diaphyseal tibial fractures. They did not state whether fractures were non-displaced but fracture configuration included two short oblique and three comminuted tibial diaphyseal fractures. All fractures had bony union after 4 weeks on follow-up radiographs. One iatrogenic fibular fracture occurred during pin placement but did not affect outcome and the fibula had evidence of bony union radiographically at follow-up. One patient had evidence of partial proximal tibial growth plate closure at fracture healing. All patients were reported to have no evidence of lameness when trotting based on owner telephone questionnaires 1–2 months after ESF removal. All five cases were considered very good to excellent outcomes treated with ESF in this case series which allows the reader the cautiously consider this method as a potential technique for non-displaced tibial diaphyseal fractures in skeletally immature dogs. Limitations of this study include its retrospective nature with low case numbers, sitting low on the hierarchy of evidence.

Complication rates for the use of ESF in dogs a retrospective study by Beever et al. (2018) report a fixator-associated complication rate of 67/97 (69%) when used in a variety of locations of the appendicular skeleton where the case population age ranged from 2 months to 13 years. Pin tract infection was recorded in 38/97 (39%) of cases. Only skeletally mature dogs with diaphyseal tibial and fibular fractures were included in this study. Tibia made up 17/97 (17.5%) of the case number in this study. Interestingly, the complication rate for tibial fractures were 7/17 (41%) where deep pin tract infection was the most common. Again, the retrospective nature of this study and the small numbers of tibial fractures included, makes drawing of any firm conclusions from the data published by the authors limited. Although tibial fractures were included in this study, they made up less than 18% of the case population. The reader should appreciate the types of complications discussed such as superficial or deep pin tract infection, implant failure and bone fracture are possible to occur in non-displaced tibial and fibular fractures in skeletally immature dogs stabilized with ESF. Beever et al. (2018) discuss that pin tract infection is likely due to bacterial colonisation at the skin-pin interface where bacteria can form biofilms and avoid host immune response and antimicrobial therapy. The advantage of implant-associated infections in the context of ESF is that these should resolve after explantation, which is always pre-planned. Removal of ESF implants is less involved than removal of internal implants. It is important that the veterinarian recognises that pin tract infection is common and that appropriate communication with the owner is required to make them aware of the signs associated with pin tract infection, as antibiotic therapy may be required if infection occurs.

Complications with regards to internal fixation in companion animals has been well reported in the veterinary literature. Vallefuoco et al. (2016) reported complications for locking compression plates (LCP) used for appendicular fractures in dogs and cats where the study populations age ranged from 2 months to 18.3 years. Their overall implant related complication rate was 7/75 (9%), interestingly 57% of the complications noted (4/7 implant related complications) affected the tibia. Implant related complications included plate breakage, plate bending, screw pull out, screw fracture. Non-implant related complications included wound related (dehiscence) and osteomyelitis. However, this study did not aim to solely focus on diaphyseal tibial and fibular fractures or specifically skeletally immature patients making it less relevant to the PICO question in focus. Nonetheless, the complications described should be considered as possible by veterinarians willing to use plate fixation when approaching non-displaced tibial diaphyseal fractures in skeletally immature dogs. Veterinary surgeons must require a level of preparedness to deal with these complications should they occur.

In a retrospective study by El-Shafey et al. (2022) looking at tibial and fibular fractures in 47 dogs and 35 cats, 30/82 (37%) were dogs under 1 year old. 39/82 (47.6%) of the total case population were treated with open reduction and internal fixation, with 15/82 (18.3%) of these being repaired with plates and screws. The remainder were fixed with intramedullary pins and cerclage wire only. 11/82 (13%) of internally fixed tibial fractures had complications such as malunion, implant failure, and osteomyelitis, highlighting again that internal fixation is not devoid of complications. The practicalities and postoperative recovery of internal versus external fixation should always inform the veterinarian’s decision when considering these two different approaches.

In order to address this PICO, a retrospective study comparing specifically non-displaced diaphyseal tibial and fibular fractures in skeletally immature dogs would be required to collect data regarding postoperative complications.

To obtain valid evidence comparing healing between internal and external fixation, a randomised controlled prospective study would be required, where non-displaced fractured tibiae and fibulae are randomly assigned a fixation method and radiographed regularly during recovery to assess the extent of healing and bony union using a radiographic grading system (Hammer et al., 1985). Postoperative complications would then be screened for and recorded prospectively until long-term follow-up could be obtained (e.g., over the course of the first 12 months postoperatively). This paper highlights how, unsurprisingly, prospective recording of complications is much more accurate than retrospective recording (Turk et al., 2015). Even with this study design, diaphyseal tibial and fibular fracture configuration and cause of trauma could affect healing due to damage to the soft tissue envelope, which would pose challenges for retrieving accuracy of comparison. Similar fracture configurations could be compared with each method as used by Beever et al. (2018). Within each method of fixation, some standardisation of plate type and ESF type would be required for more valid comparison as there is such a variety of implants available for IPF and ESF. Furthermore, not all fracture configurations may be appropriate for either fixation method with this study design.

In conclusion, there is currently no evidence that skeletally immature dogs with non-displaced diaphyseal tibial and fibular fractures have a better outcome when treated with internal fixation versus external fixation.

## Methodology

**Search strategy:** 

Databases searched and dates covered:	CAB Abstracts on OVID Platform 1973–week 32 of 2023 PubMed on the NCBI interface 1920–week 32 of 2023
Search strategy:	CAB Abstracts: (dog or dogs or puppy or puppies).mp. or exp puppies/ or exp dogs/ (tibia* and fibula* and fracture*).mp. ((fixat* or stabilis* or stabiliz*) and (external or internal)).mp. 1 and 2 and 3 PubMed: dog OR dogs OR puppy OR puppies (tibia AND fibula AND fracture (fixation OR fixator OR stabilise OR stabilize) AND (external OR internal) 1 AND 2 AND 3
Dates searches performed:	14 Aug 2023

**Exclusion / inclusion criteria:** 

Exclusion:	Only one technique included. Dogs that were skeletally mature. No postoperative follow-up included. Articles not available in English. Book chapters. Single case reports. Congress proceedings. Expert opinions. Articles irrelevant to the PICO.
Inclusion:	Prospective and retrospective studies. Comparing outcomes of skeletally immature dogs with external or internal fixation for diaphyseal tibial and fibular fractures. Objective comparative assessment of fracture healing.

**Search outcome:** 

Database	Number of results	Excluded – Not relevant to PICO question	Total relevant papers
CAB Abstracts	25	25	0
PubMed	13	13	0
Total relevant papers when duplicates removed			0

## ORCiD

Jake Chitty: https://orcid.org/0000-0001-7011-006X

Paul Aldridge: https://orcid.org/0000-0002-3805-2610

## Conflict of interest

The authors declare no conflicts of interest.
